# Cross‐sectional survey of cattle haemopathogens in Constantine, Northeast Algeria

**DOI:** 10.1002/vms3.459

**Published:** 2021-03-08

**Authors:** Asma Amina Foughali, Hocine Ziam, Asma Aiza, Halima Boulkrout, Ali Berber, Idir Bitam, Mohamed Gharbi

**Affiliations:** ^1^ Laboratoire de Biodiversité et Environnement : Interactions et Génomes. Université des Sciences et de la Technologie Houari Boumédiène Algiers Algeria; ^2^ Institut des Sciences Vétérinaires Université Saad Dahlab, Ouled Yaich Blida Algeria; ^3^ Laboratoire de Biotechnologie Environnement et Santé Université Saad Dahlab Blida Algeria; ^4^ Laboratoire des Biotechnologies Liées à la Reproduction Animale (LBRA), Université Blida 1 Blida Algeria; ^5^ Private Veterinary Surgeon Beni Hamidéne Algeria; ^6^ École Supérieure en Sciences de l’Aliment et des Industries Agroalimentaires (ESSAIA), El Harrach Alger Algeria; ^7^ Laboratoire de Parasitologie École Nationale de Médecine Vétérinaire de Sidi Thabet Univ. Manouba Sidi Thabet 2020 Tunisia

**Keywords:** Algeria, *Anaplasma marginale*, *Babesia bovis*, Bovine, *Theileria annulata*

## Abstract

This aim of the present study was to estimate the prevalence of haemopathogens in cattle in Beni Hamidene locality, district of Constantine (Νortheastern Algeria). Between June and October 2014, 169 bovines from 25 farms were included in this survey, 32 (18.9%) among them were suspected of piroplasmosis and/or anaplasmosis. Infection prevalences were estimated by microscopic examination of Giemsa‐stained blood smears and blood samples from all included cattle (*n* = 169). Animals were infected by *Theileria annulata* (65/169; 38.46%), *Anaplasma marginale* (22/169; 13%) and *Babesia bovis* (5/169; 3%). Two co‐infection patterns were found: *Theileria annulata/Anaplasma marginale* (7.69%) and *Theileria annulata/Babesia bovis* (1.18%). Only one farm had no cattle infected by any of the haemopathogens. There was a signification difference of *T. annulata* infection prevalence according to age category (*p* =.04). These results emphasised mainly the presence of bovine tropical theileriosis in northeastern, Beni Hamidene locality, province of Constantine, Algeria.

## Graphical abstract

This aim of the present study was to estimate the prevalence of haemopathogens in cattle in Beni Hamidene locality, district of Constantine (eastern Algeria)

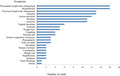

## 1 INTRODUCTION

Piroplasmosis and anaplasmosis are non‐contagious vector‐borne diseases transmitted by several haematophagous arthropods. Bovine tropical theileriosis is a protozoan disease caused by *Theileria annulata* transmitted by ticks of the genus *Hyalomma*, affecting lymphocyte and then red blood cells. (Bilgic et al., 2019; Dolan, 1989; Nourollahi‐fard et al., 2015). Babesiosis is haemoprotozoan diseases caused by the presence and multiplication of *Babesia* spp. in erythrocytes (Bock et al., 2004; Bouattour et al., 2004). They are transmitted by several ixodid ticks, mainly *Rhipicephalus* (*Boophilus*) (Barré & Camus, 1983; Uilenberg, 2006). *Babesia divergens* is transmitted by *Ixodes ricinus* (Zintl et al., 2003) but was never reported in Algeria. *Babesia bovis* infection is characterised by fever, icterus, anaemia, haemoglobinemia, haemoglobinuria and respiratory and nervous symptoms (Everitt et al., 1986; Otgonsuren et al., 2020).

*Anaplasma marginale* causes bovine anaplasmosis (Ben Said et al., 2018; Yang et al., 2017); it is an intracellular ricketsial organism (Rar & Golovljova, 2011), transmitted mechanically by biting flies and biologically by ticks (Scoles et al., 2008). Symptoms of anaplasmosis include fever, anaemia, icterus, weight loss, abortion (Kocan et al., 2003; Aktas and Özübek, 2017). These three infections cause important economic losses in cattle (Rahali et al., 2014; Uilenberg, 1995).

Four techniques are used for diagnosis of haemopathogens: (1) Giemsa stained blood smears is the quickest and the cheapest technique. It allows the estimation of the parasitaemia and detects the presence of any co‐infection by haemopathogens. It allows also the detection of leukocytes infected by *T. annulata* schizonts in lymph‐node biopsies (Bilgic et al., 2016). However, it has low sensitivity in detecting carrier animals (Alvarez et al., 2019; Ashuma et al., 2013; M’ghirbi et al., 2008; Uilenberg, 2004). (2) Enzyme Linked Immunosorbent Assay (ELISA) is used to detect specific antibodies (Al‐Hosary et al., 2015, 2020); it has a high sensitivity and specificity (Santamaria et al., 2020) and many samples can be easily tested (Salih et al., 2007). As ELISA becomes positive approximately 3 weeks after infection, this technique is only used for epidemiological studies to detected carrier animals with low parasitaemia. (3) The indirect immunofluorescent antibody test (IFAT) is also used to detect carrier animals (Nayel et al., 2012). This technique is very time‐consuming and, in some cases, the interpretation of the fluorescence is difficult.

(4) Several PCR techniques are used for the detection of haemopathogens (conventional PCR, nested PCR, Real‐time PCR, PCR multiplex, PCR‐RFLP, reverse line blot); they have high sensitivity and specificity (Wang et al., 2019) but they are relatively expensive (Liu et al., 2014). Moreover, these techniques don't differentiate between carrier and clinically infected animals.

In Algeria, tick‐borne diseases represent a real constraint for cattle owners. For example, the mean milk yield decreases to 319 L/cow that suffers tropical theileriosis during 2 months following the infection (Benchikh Elfegoun et al., 2017). Ayadi et al. (2016) estimated that the mean daily milk yield decreases during 2 months to 2.76/day/cow presenting clinical theileriosis.

The aim of the present study was to estimate the prevalence of piroplasmosis and anaplasmosis in cattle, in Beni Hamidene locality, province of Constantine, Northeast Algeria.

## 2 MATERIALS AND METHODS

### 2.1 Study area

**FIGURE 1:**
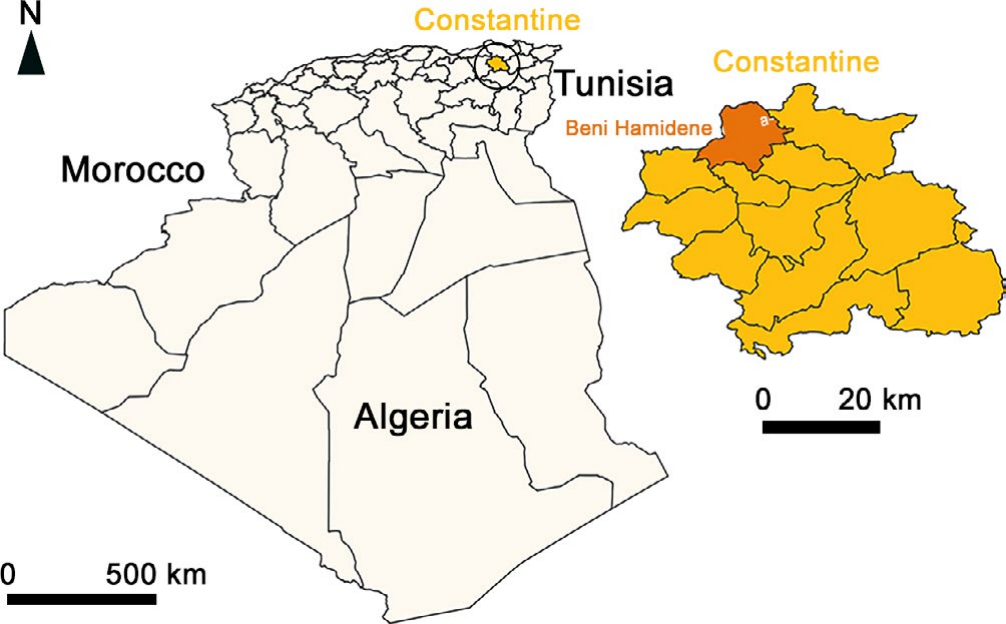
Geographical location of Beni Hamidene locality, Wilaya of Constantine, Algeria

The present survey was carried out in Beni Hamidene locality (36°30' S; 6°31'W) located in northwest of Constantine district (Northeast Algeria) and has 131 km^2^ area (Figure 1). Beni Hamidene has an altitude that varies between 300 and 1,364 m, a sub‐humid and semi‐arid climate and a mean annual pluviometry of 761 mm. There are 171 cattle breeders in Beni Hamidene, owning approximately 1,640 animals, among them were 783 dairy cows (Direction des Services Agricoles de la Wilaya de Constantine, 2015).

### 2.2 Characteristics of studied farms and animals

A cross‐sectional study was carried out between June and October 2014 in 25 cattle farms containing between 2 and 40 cattle (average population per farm: 10 cattle). Among the 248 cattle in these farms, 169 were included in this study. Animals that received piroplasmicides during the 2 months before our visit were excluded from the study. Almost half of the farms were dairy (12/25) or mixed (12/25), and there was one beef cattle breeder. All farms except one were managed under a semi‐intensive system (24/25). The majority of farms (24/25) had cracked walls and were poorly maintained (16/25). More than half of them (16/25) were housing cattle with other animal species; the majority of cattle were crossbreed (76%; 129/169), followed by the Atlas Brown breed (15%, 26/169) and the exotic pure breeds (Holstein, Frisian, Montbeliarde and Charolaise) (8%; 14/169), sampled cattle were aged between 3 months and 13 years (mean age: 3.7 years). The majority of cattle were females (85%; 143/169) (sex‐ratio M : F = 0.18). Among the 25 surveyed farms, 16 were using acaricides on all cattle when at least one animal in the farm was infested by ticks. Fifteen farmers prefer flumethirn (Bayticol 1%^®^, Bayer, Germany), and one uses foxim (Sebacil 50%^®^, Bayer, Germany).

### 2.3 Sample collection

Among the 169 studied cattle, 32 (18.9%) presented clinical signs of piroplasmosis and/or anaplasmosis (anaemia, icterus, ocular /vaginal petechiae, hyperthermia, swelling of lymph nodes, apathy, anorexia, hypogalactia and weight loss) The animals were clinically examined and blood samples were collected from the ears capillaries with sterile disposable scalpels. Blood smears were done in the farm and immediately fixed in 100% methanol for 3 min, air‐dried and then transported to the laboratory. Blood smears were Giemsa‐stained for 10 min, air‐dried and then rinsed with tap water and air‐dried. For each slide, 50 microscope fields were examined at 1000X magnification under microscope with immersion oil. The infection intensity by haemopathogens was semi‐quanlitatively estimated, and animals were ranked into four categories: low (0.2–1 parasites/microscopic field), mild (1–5 parasites/microscopic field) and high (5 > parasites/microscopic field).

### 2.4 Treatments

All cattle suspected of piroplasmosis and/or anaplasmosis (32/169) were treated. Buparvaquone (Butacof 5^®^ *,* Boehringer Ingelheim, Germany) was injected intramuscularly at the conventional dose of 2.5 mg/kg to cattle with tropical theileriosis clinical signs.

Animals with anaplasmosis received oxytetracycline (Longicine^®^, Vetoquinol, France) at the conventional dose of 20 mg/kg and anti‐anaemic (Fercobsang^®^, Vetoquinol, France) at the dose of 217 and 435 mg per young and adult cattle, respectively.

Imidocarb (Imidocarb‐LH^®^, Vetopharm, Algeria) was injected to animals with babesiosis at the dose of 3 mg/kg. Those presenting fever (63%; 20/32) received phenylbutazone (Butasyl^®^, Zoetis, USA) at the dose of 1–5 mg/kg. Additionally oral drenching of Rumicen Poudre Complex (Cenavisa, Spain) was administered to animals with digestive disorder.

### 2.5 Statistical analyses

Infection prevalences were compared using either chi‐square test or stratified Mantel‐Haenszel chi‐square test at 0.05% threshold with Epi info 2000 software (Schwartz, 1993).

## 3 RESULTS

Among the 25 visited farms, 13 contained at least one cattle infected by one haemopathogen, 11 farms contained both single and co‐infection of haemopathogens and one farm had non‐infected cattle.

Giemsa‐stained slides examinations revealed that 46% (77/169) were infected by at least one of the three haemopathogens. *Theileria annulata* (38%; 65/169) was the most frequent pathogen followed by *Anaplasma marginale* (13%; 22/169) and *Babesia bovis* (3%; 5/169) (*p* < .05). Two co‐infection patterns were found: *Theileria annulata/Anaplasma marginale* (8%; 13/169) and *Theileria annulata/Babesia bovis* (1%; 2/169). Infection prevalence by *T. annulata* was significantly higher than *A. marginale* (*p* =.033) (Table 1). Almost half of *T. annulata‐*infected cattle (48%; 31/65) had a mild parasitaemia [0.33%–1.67%]. The high and low intensities of *Theileria annulata* in infected erythrocytes were 13.85% (9/65) and 38% (25/65), respectively. All infected cattle (22/22) by *Anaplasma marginale* and *Babesia bovis* (5/5) had low infection intensity.

**TABLE 1:** Characteristic of farms and infected cattle by haemopathogens in Beni Hamidene locality, Wilaya of Constantine, Algeria

Epidemiological characteristics	*Theileria annulata*	*Babesia bovis*	*Anaplasma marginale*
Farms	Number of farms with at least one infected cattle/number of visited farms		
Dairy farms	11/12	3/12	3/12
Mixed farms	10/12	2/12	11/12
Beef cattle farms	1/1	0/1	0/1
Promiscuity with other animal species	14/16	4/16	10/16
Cracks in walls	21/24	5/24	14/24
*Total*	22/25	5/25	14/25
Animals	Number of infected cattle/number of examined cattle (% ± SE)		
*Sex*			
Males	8/26 (31 ± 18)	0/26	3/26 (12 ± 12)
Females	57/143 (40 ± 8)	5/143 (3 ± 3)	19/143 (13 ± 6)
*Age*			
<1 year	5/18	0/18	0/18
[1 – 2 years [	12/40 (30 ± 14.2)	1/40 (2.5 ± 4.8)	5/40 (12.5 ± 10.3)
≥2 years	48/111 (43.24 ± 9)	4/111 (14.41 ± 6)	17/111 (15.32 ± 7)
*Breed*			
Exotic pure breeds	7/14	1/14	4/14
Crossbreed	49/129 (37.98 ± 8)	4/129 (3.1 ± 3)	14/129 (10.85 ± 5)
Atlas Brown breed	9/26 (34.61)	0/26	4/26 (15.38)
*Total*	65/169 (38.46 ± 7)	5/169 (2.96 ± 3)	22/169 (13.02 ± 5)

Abberivation: SE: Standard error

More than half of the visited farms (14/25) had at least one cattle infected by *A. marginale*. All the *A. marginale‐*infected cattle (*N* = 22) were not infested by ticks. Only one animal had symptoms of anaplasmosis and three co‐infections by *Anaplasma marginale*/*T. annulata*. *A. marginale‐*infected cattle were aged of 1 year and more, and 17 cattle were aged of 2years and more (Table 1). *A. marginale* infection was observed in 3 males and 19 females out of 26 and 143, respectively (*p* >.05).

The majority of farms (22/25) had at least one cattle infected by *T. annulata*, among the 32 suspected animals of piroplasmosis and/or anaplasmosis, 25 (78%) showed tropical theileriosis symptoms.

Infection prevalences in mixed farms were 10/12, 2/12 and 11/12 for *T. annulata*, *B. bovis* and *A. marginale*, respectively. Whereas, in dairy farms, infections prevalences were 11/12, 3/12 and 3/12 for *T. annulata*, *B. bovis* and *A. marginale*, respectively.

More than half of *T. annulata‐*infected cattle (58%; 38/65) were 3 years old or more, 8% (5/65) were calves of less than 1 year old and 34% (22/65) were aged between 1 and 3 years old (*p* =.04). Infection prevalence did not significantly vary according to sex (*p* >.05). Infestation prevalence by ticks in *T. annulata*‐infected cattle was estimated to 4.6%, almost all *T. annulata‐*infected cattle (95.39%; 62/65) were not infested by ticks. There was no significant difference in infection prevalence according to breeds (*p* =.62).

**FIGURE 2:**
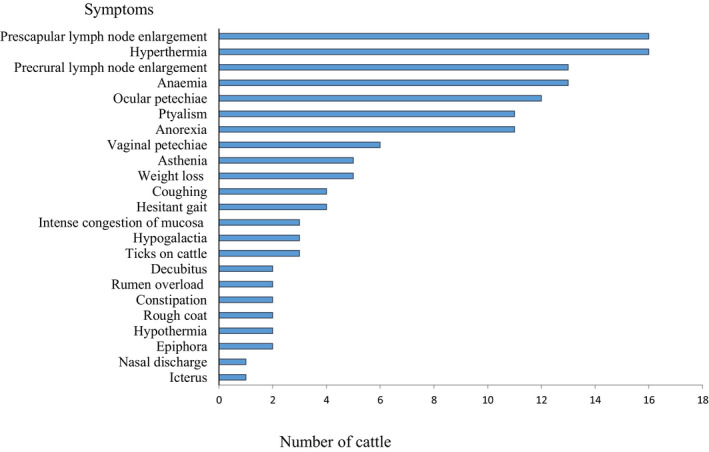
Number of tropical theileriosis symptoms observed in 25 cattle with tropical theileriosis in Beni Hamidene locality, Wilaya of Constantine, Algeria

In the visited farms, cattle infected with theileriosis showed symptoms of fever and prescapular lymph node enlargement (16/25), followed by anaemia (13/25), hypersalivation (11/25), coughing (4/25), intense congestion of mucosa (3/25), constipation (2/25) and icterus (1/25) (Figure 2).

Few farms (5/25) had one *Babesia bovis‐*infected cattle. Among the 169 examined cattle, 5 (3%) were infected by *B. bovis*. The five *B. bovis‐*infected cattle were at least 1.5 years old and were not anaemic. Two out of the 5 *B. bovis‐*infected cattle were infested by ticks.

Among the 32 clinically suspected animals of piroplasmosis and/or anaplasmosis, the morbidity, lethality and mortality rates were 18.9% (32/169), 6% (2/32) and 1.2% (2/169), respectively.

## 4 DISCUSSION

The majority of the studied farms contained at least one cattle infected by *T. annulata*, this could be explained by the presence of cracks in almost all the visited farms, which are favourable for the hibernation of nymphs and laying eggs by *Hyalomma scupense* female ticks, the vector of *T. annulata* in Maghreb region (Gharbi et al., 2014). However, in our study only three of the infected cattle (3/32) were infested by ticks which could because of the use of acaricides by the majority of cattle owners (16/25) and the long delay between tick fixation and clinical symptoms occurrence, generally, the bloodmeal duration of ticks is shorter than the incubation period, that's why, when the animal shows clinical symptoms, the tick that transmitted the parasite could not be found attached to the animal. However, farmers do not use acaricides properly (concentrations of acaricides and/or interval between two applications were not corrected). The low tick numbers and the presence of clinical cases indicate that the visited farms were in an endemic instability state for tropical theileriosis.

In endemic regions, carrier state prevalence is high, it can even reach 100%. Giemsa‐stained blood smears lacks sensitivity because the parasitaemia in these animals is usually low (Alvarez et al., 2019; Gharbi et al., 2012).

Infection prevalence of *Theileria annulata* was higher than *A. marginale* and *B. bovis*. The same trend was reported by Ait Hamou et al. (2012) in Morocco.

In Annaba and El Taref (Northeast Algeria), the infection rates in diseased animals were 53.7%; 7.4%, and 5.6% for *T. annulata*, *A. marginale* and for *B. bovis,* respectively (Ziam & Benaouf, 2004). In central Algeria, 36.9%; 3.4% and 4.1% of the examined cattle were infected by *T. annulata*, *B. bovis* and *A. marginale*, respectively (Ziam et al., 2017). In Boutheldja region (Wilaya of El Taref), *B. bovis*, *T. annulata* and *A. marginale* were reported in 33.3%; 47.6% and 40.5% of examined cattle, respectively (Dib et al., 2008).

*Theileria annulata* infection prevalence (38%) was comparable to that reported in Central Algeria (37%) (Ziam et al., 2017), and slightly higher than that reported in the State of Chhattisgarh in India (23%) (Naik et al., 2016).

Clinical signs observed in 32 sick animals were typical of bovine tropical theileriosis; 78% (25/32) were infected by *T. annulata* and similar to those reported by other authors (Khatoon et al., 2013; Muhammed et al., 1999; Ziam et al., 2016). However, in the survey conducted by M’ghirbi et al. (2008) in autumn after the disease season, *T. annulata*‐infected cattle (17.3%; 48/278) did not showed symptoms because animals were carriers. However, our study was conducted during the disease season, summer. Benchikh Elfgoun et al. (2017) examined 89 cattle clinically infected by piroplasms in two provinces in northeastern Algeria (Skikda and Oum Bouaghi), they found that 94 and 33.7% of them were infected by *T. annulata* and *B. bovis*, respectively.

The majority of cattle with clinical signs were infected by *T. annulata* (78.13%; 25/32), including three cattle co‐infected by *T. annulata* and *A. marginale* and one by *T. annulata* and *B. bovis*. One cattle was infected by *A. marginale* and another by *B. bovis*.

Half of tested exotic breed cattle (7/14) were infected by *T. annulata*. However, the absence of a significant prevalence (*p* >.05) between breeds was probably because of the small sample size of cattle. Among the169 cattle, 4/14; 14/129; 4/26 were infected by *A. marginale* in exotic pure breeds, Crossbreed and Atlas Brown breed, respectively. All cattle breeds were infected by *A. marginale*, similarly AL‐Hosary et al. (2020) and Ait Hamou et al. (2012) showed that *A. marginale* prevalence did not vary according to cattle breed.

Among the 26 tested Atlas Brown cattle, 9 were carriers confirming that the local breed is more resistant to haemopathogens (Ait Hamou et al., 2012; Glass & Jensen, 2007; Saleem et al., 2014). However, there was no significant difference in infection prevalence according to breeds. Carrier local cattle breeds are sources of infection for other animals, mainly susceptible breeds (Moni et al., 2019).

The majority of *A. marginale*‐infected cattle were carriers. This result is similar to that reported by M’ghirbi et al. (2016), who found in Tunisia that all *A. marginale‐*infected cattle were carriers. This infection persisted for the whole life of cattle (Aubry & Geale, 2011; Kocan et al., 2010). All *A. marginale*‐infected cattle were more than 1‐year age, the similar trend was also reported in *B. bovis‐*infected cattle. According to Kocan et al. (2003), calves are less susceptible to *A. marginale* infection. This can be explained by the presence of maternal antibodies (Abdela et al., 2017) and the lower attractivity of young cattle to ticks (Gharbi et al., 2013). In our study, there was a positive correlation between infection prevalence and age. Indeed, *T. annulata* infection prevalence in cattle aged of 3 years and more was significantly higher (58%) than the prevalence in cattle aged of less than 3 years (42%; *p* =.04). This can be explained by the stress caused by lactation that induces immunodepression and the presence of higher tick burdens when compared with young animals (Gharbi et al., 2013). According to Yessinou et al. (2018), calves are less infested than adult cattle by ticks. However AL‐Hosary et al. (2018) reported that cattle aged less than 1 year were more susceptible to infection by *T. annulata* (83%; 400/480). According to AL‐Hosary et al. (2018) cattle acquire immunity with age and after multiple infections by *T. annulata*. In our study, cattle probably have not acquired immunity; this can be explained by the introduction of naive cattle in the farms and the low tick burdens because of acaricides application.

Infection prevalence by *B. bovis* (3%; 5/169) was slightly lower than that reported by Ziam et Benarouf (2004) in eastern Algeria (5,6%). According to Ziam et al. (2017), the low rate of *B. bovis* is because of the low tick burdens in the studied region and to the relatively low pasturing duration. In addition, it can be explained by the fact that Beni Hamidene has a sub‐humid and semi‐arid climate which is not suitable for development of the ticks vector *Rhipicephalus* (*Boophilus*) *annulatus*. According to Benchikh‐Elfegoun et al. (2007), this tick species is adapted to humid climate. However, according to Calder et al. (1996) parasitaemia fluctuation in *B. bovis* chronicallyinfected cattle could explain that the low detection of parasites ranges from 1. 10^–5^ to 1. 10^–7^. The prevalence found in the present study was lower than in Syria (15.46%; Terkawi et al. 2012), Mongolia (18%; Battsetseg et al. 2018) and in South Africa (35.3%; Terkawi et al., 2011). The difference can be explained by the diagnostic method (ELISA), which has a higher sensitivity than Giemsa‐stained blood smears of carrier animals (Guswanto et al., 2017).

In the present study, the prevalence of co‐infections by *T. annulata* and *B. bovis* (1.2%) was similar to that reported by Ziam et al. (2017) (1.1%). The prevalence of co‐infections by *T. annulata* and *A. marginale* (7.69%) was slightly higher than that reported by Ziam et al. (2017) (1.9%) in north‐central Algeria. This could be because of the difference in *Anaplasma* spp. vector ecology, particularly its biomass and typology in the two regions, which have, as far as we know, never been studied.

All *B. bovis*‐infected cattle (5/5) were aged of 1 year and more. Young cattle are more resistant to babesiosis than adult (Ekici & Sevinc, 2009; Goff et al., 2001). In addition, calves are less exposed to the vectors of *Babesia* because *R. (Boophilus) annulatus* are found in the pastures where generally older cattle grazes.

This study showed the presence of three haemopathogens in cattle in Beni Hamidene locality, district of Constantine, Algeria. Among these haemopathogens, *Theileria annulata* was the most frequent. This study also reported the main clinical features of theileriosis. No Atlas Brown cattle showed symptoms, as a local breed, breeding Atlas Brown cattle must be encouraged in the farms where control of vector ticks is difficult to implement. In addition, extension programmes must be implemented for Algerian breeders to optimise the acaricide application.

Moreover, farmers must be sensitised on the importance of sustainable control options (cleaning, wall roughcasting and smoothing) to reduce the shelters for *Hyalomma scupense* off‐host stages.

## CONFLICT OF INTEREST

All the authors declare that they have no conflicts of interest with the work presented here.

## AUTHOR CONTRIBUTION

**Asma Amina Foughali:** Conceptualization; Data curation; Formal analysis; Funding acquisition; Investigation; Methodology; Resources; Software; Supervision; Validation; Visualization; Writing‐original draft; Writing‐review & editing. **Hocine Ziam:** Validation; Visualization. **Asma Aiza:** Methodology. **Ali Berber:** Project administration. **Idir Bitam:** Project administration. **Halima Boulkrout:** Conceptualization; Investigation; Methodology; Supervision; Visualization. **Mohamed Gharbi:** Conceptualization; Data curation; Formal analysis; Methodology; Project administration; Software; Supervision; Writing‐review & editing.

### Peer Review

The peer review history for this article is available at https://publons.com/publon/10.1002/vms3.459.

## Data Availability

The datasets generated during the current study are available from the corresponding author on reasonable request.
